# Maternal views on facilitators of and barriers to breastfeeding preterm infants

**DOI:** 10.1186/s12887-018-1260-2

**Published:** 2018-08-27

**Authors:** Maria Lorella Gianni, Elena Nicoletta Bezze, Patrizio Sannino, Michela Baro, Paola Roggero, Salvatore Muscolo, Laura Plevani, Fabio Mosca

**Affiliations:** 1 Fondazione I.R.C.C.S. Ca Granda Ospedale Maggiore Policlinico, Neonatal Intensive Care Unit, Department of Clinical Science and Community Health, University of Milan, Via Commenda 12, 20122 Milan, Italy; 2 Fondazione I.R.C.C.S. Ca Granda Ospedale Maggiore Policlinico, S.I.T.R.A. Basic Education Sector, Via Francesco Sforza 28, 20122 Milan, Italy

**Keywords:** Preterm infants, Breastfeeding, Maternal experience

## Abstract

**Background:**

The supply of breast milk to preterm infants tends to occur at a lower rate than that recorded among term infants. We aimed to investigate the facilitators of and barriers to breastfeeding during hospital stay according to the experiences of mothers that gave birth to premature infants requiring admission to neonatal intensive care unit.

**Methods:**

A cross-sectional questionnaire survey was conducted. Mothers who had delivered a newborn with a gestational age ≤33 weeks requiring intensive care, entered the study. Basic subjects’ characteristics and infant feeding practices were also recorded.

**Results:**

A total of 64 mothers were enrolled, leading to a total of 81 infants. At discharge, any breastfeeding was recorded in 66% of infants, with 27% of those infants being exclusively breastfed. Any infant was exclusively fed directly at the breast. Most mothers experienced adequate support during their infant’s hospitalization and reported satisfaction with breastfeeding. Almost all mothers felt that feeding their infant human milk was beneficial for the infant’s health. Thirty percent of the mothers reported that they had experienced some obstacles to breastfeeding. Specifically, infants born to mothers who experienced difficulties in pumping breast milk (OR = 4.6; CI 1.5–13.9) or in providing an adequate amount of milk to the infant (OR = 3.57; CI 1.1–11.5) were at higher risk of being fed with formula at discharge.

**Conclusions:**

On the basis of the present results, health care professionals should target their efforts to optimize breastfeeding support for mothers of premature infants admitted to level III care, especially by improving breast milk production and endorsing direct breastfeeding.

## Background

Human milk is the best nutrition for all infants, including preterm infants [1]. Although human milk-fed preterm infants receive unique health benefits, such as reductions in prematurity-associated complications and promotion of their long-term development [2], rates of successful breastfeeding among infants born prematurely tend to be lower than in term infants [3]. Indeed, admission to the neonatal intensive care unit (NICU) represents a highly challenging context due to the separation of the mother from the infant, the difficulties encountered by the mothers in initiating and maintaining milk production and the need for dealing with hospital staff in a high technology environment [4]. Accordingly, support, protection and promotion of breastfeeding in the NICU have been advocated. Maternal education about providing human milk, reinforcement of maternal motivation, support of parents’ roles, and motivation and education of health care professionals have been identified as the major factors that support breastfeeding in NICUs [5, 6].

Family centred care, which aims to involve families in taking care of their infants and in the decision process regarding their infant’s care, contributes to optimization of neonatal care [7]. Indeed, the experience of the mother in supplying human milk is a unique opportunity for being close to the infant and being involved in his/her care during a NICU stay [8]. Previous studies addressing breastfeeding experience of preterm mothers report that milk expression and breastfeeding are perceived as a way to reconnect with their infant and empower the development of motherhood. Nonetheless these tasks result to be demanding and unfamiliar. Moreover, the expression of inadequate amount of milk leads to feelings of failure and frustration [9].

Within this context, there is a need to investigate and gain further insight into mothers’ experiences regarding breastfeeding during their hospital stay in order to optimize interventions aimed to increase their knowledge and engagement [5].

We aimed to investigate the facilitators of and barriers to breastfeeding during hospital stay according to the experiences of mothers that gave birth to infants born prematurely and requiring admission to neonatal intensive care unit.

## Methods

### Design and setting

We conducted a cross-sectional survey study from January to June 2016 among mothers whose infants required admission to the Neonatal Intensive Care Unit (NICU) and/or the Medium Care of Authors’ Institution. The hospital covers almost 6000 pregnancies per year and offers tertiary care services for complicated pregnancies. The NICU capacity is 23 beds whereas that of the Medium Care is 33 beds.

The feeding practices of the institution of this study are described in details elsewhere [10]. Lactation was promoted and supported. Specifically, mothers were informed on the beneficial effects of human milk feeding; they were then instructed on how to initiate and maintain a high frequency of milk pumping per day. When infants were in stable condition, mothers were supported to have their infants put at breast, even for a limited period of time. If human milk was not available or in short supply, formula milk was provided.

The study was approved by the institutional Ethical Commitee. Written informed consent was signed by the mothers with regard to the completion of the survey and by both parents for the collection of infants’ medical data.

### Sample

Mothers were recruited using convenience sampling. Inclusion criteria required that participants were mothers that had delivered newborns requiring intensive care, with a gestational age ≤33 weeks and spoke Italian as their native language. Exclusion criteria were the presence of medical contraindications for breastfeeding, mothers who are not intending to breastfeed and mothers of newborns that died during the hospital stay or were transferred to another facility.

### Data collection procedures

Mothers were asked to take part in the study by the investigator 24-h before infants’ hospital discharge The mothers took approximately 15–20 min to complete the self-administered questionnaire.

### Instruments

A revised version of the original tool [11] was used to assess the facilitators of and the barriers to breastfeeding: specifically, items investigating the availability of lactation support in NICU, whether the mothers knew someone who had a previous experience of breastfeeding, whether someone from the family group had breastfed, how long the mothers had been breastfeeding their infant were removed. The items exploring how long mothers had planned to breastfeed, whether the mothers had taken a breastfeeding class and the elements on which mothers based their decision either to breastfeed or bottle feed their infant were also removed.

In order to investigate the local hospital policies, six items (*n* = 11–13 and *n* = 17–19) were introduced. A further item (*n* = 14) investigating maternal previous breastfeeding experience was also added.

The back-translation method was adopted to translate the scale into Italian. Before beginning the survey, the Italian version of the revised tool, which includes 19 items, was administered to a sample of mothers who had newborns admitted to the NICU; this was done in order to highlight any issues in the items comprehension. The first 10 items investigated the mother’s breastfeeding experience: mothers were required to rate their degree of agreement for each item on a 4 point Likert scale, ranging from “Strongly Disagree” on one end to “Strongly Agree”. The subsequent three items investigated when the mothers were first supported by a lactation consultant/breastfeeding counsellor, when the mothers began producing their milk and how many times per day mothers produced their milk (items 11–13). Mothers were then asked about their previous breastfeeding experience, whether the decision to breastfeed their infant was made during pregnancy or after delivery, and whether advice regarding breastfeeding had been given during pregnancy by health care professionals (items 14–16). Items 17–19 investigated whether the mothers were given any information on infant’s hunger indicators and the quality of the infant’s suckling at the breast and how human milk was first given to the infant (i.e., directly supplied or supplied by bottle). There were also two additional open-ended questions, enquiring about the barriers to and facilitators of breastfeeding that the mothers had experienced during hospitalization.

Maternal sociodemographic characteristics and variables related to pregnancy were recorded. Data collection on basic infants’ characteristics and hospital stay, including the occurrence of any comorbidities and feeding, was also performed. Gestational age was calculated on the basis of mother’s last menstrual period and the ultra sonographic biometry. Infants were classified as appropriate or small for gestational age according to birth weight percentile values (≥10th or <10th, respectively) [12]. Comorbidities, type and mode of feeding were classified as described elsewhere [1, 13].

### Statistical analysis

The data are presented as the mean ± standard deviation (SD) or *n* (percentage). Answers to the first 10 items were categorized into two groups (agree or disagree) for analyses. Items 11, 12 and 13 were also categorized in two groups as follows: ≤8 vs >8 h (item 11), ≤24 vs >24 h (item 12) and ≤6 vs >6 times (item 13).

The data reported in the two open-ended questions were categorized by content analysis into similar items.

Associations between items on the questionnaire and the mode of feeding at discharge (being fed any human milk vs being fed formula) were assessed using bivariate binary logistic regression.

Statistical analyses were performed using SPSS version 12 statistic software package (SPSS Inc., Chicago, IL, USA).

A retrospective power analysis for a single mean in descriptive studies was also conducted based on the mean of positive answers at the questions assessing mothers’ experiences with breastfeeding (items 1–10).

## Results

In total, 70 mothers were invited to complete the questionnaire and 64 mothers agreed to participate, resulting in a total of 81 infants. All the enrolled mothers responded to the closed-ended questions of the survey but only 37 (58%) responded to the open-ended questions.

Table 1 reports the basic characteristics of the enrolled mothers-infants pairs. The mean maternal age was 34 ± 5.3 years; most interviewed mothers were married and Caucasian, with an educational level ≤13 years. Forty-eight mothers had a singleton pregnancy, whereas 16 experienced a twin pregnancy. Nearly half of the mothers were primiparous, and the majority underwent a caesarean section.

**Table 1:** Basic characteristics of the enrolled mother-baby dyads

Mothers (n = 64)	Mean ± SD
Age (years)	34.0 ± 5.3
	N (%)
Marital status	
Married	34 (53)
Unmarried relationship	37 (42)
Single parent	3 (5)
Ethnicity	
Caucasian	55 (86)
Hispanic American	6 (9)
African	1 (2)
Asian	2 (3)
Maternal education level	
≤13 years	42 (66)
>13 years	22 (34)
Spontaneous delivery	11 (17)
Multiparous	34 (53)
Singleton pregnancy	48 (75)
Infants (n = 81)	Mean ± SD
Gestational age at birth (weeks)	29.9 ± 2.6
Birth weight (g)	1357 ± 464
Length of hospital stay (days)	58.2 ± 41.8
Weight at discharge (g)	2566 ± 669
Postmenstrual age at discharge	38.2 ± 4.1
	N (%)
Multiples	33 (41)
Females	35 (43)
Appropriate for gestational age infants	50 (83)

Fourteen infants (17%) had at least one comorbidity during their hospitalization; specifically, nine infants developed bronchopulmonary dysplasia, three infants underwent major surgical procedures and two infants developed retinopathy of prematurity (grade I–II). Three infants developed more than one comorbidity.

At discharge, 16% of infants had achieved full oral feeding and were being fed partially by bottle and partially at breast, whereas 83% of infants achieved full oral feeding exclusively by bottle. One infant did not achieve feeding independency. At discharge, any breastfeeding was recorded in 66% of infants, with 27% of those infants being exclusively breastfed.

Table 2 shows the answers to items assessing the maternal experiences with breastfeeding. The mean of positive answers to items 1–10 was 79% ± 17%. Most mothers reported satisfaction with their breastfeeding experience during their hospital stay and were confident that feeding their infant with human milk exerted beneficial effects on their infant’s health. Although most of the mothers felt that the health care professionals in the NICU were supportive with regard to breastfeeding, 31% reported that they experienced some barriers to breastfeeding, and 36% that they had problems pumping breast milk.

**Table 2:** Answers to the questions investigating maternal experiences with breastfeeding

		Mothers (*n* = 64)	
		Disagree *N* (%)	Agree *N* (%)
1	I gained a sense of satisfaction with breastfeeding during my hospital stay	11 (17)	53 (83)
2	I feel that human milk feeding is beneficial for my infant	2 (3)	62 (97)
3	I experienced some barriers to breastfeeding in the NICU	44 (69)	20 (31)
4	I feel confident in sharing breastfeeding information with health care professionals	7 (11)	57 (89)
5	I perceive supportive hospital staff attitudes in relation to breastfeeding/pumping breast milk	4 (6)	60 (94)
6	I feel that the hospital staff is well educated on breastfeeding/pumping breast milk	10 (16)	54 (84)
7	I perceive that the resources available to support me with breastfeeding are appropriate	15 (23)	49 (77)
8	I experienced some difficulties in pumping breast milk	41 (64)	23 (36)
9	I experienced some difficulties in providing an adequate amount of milk to my infant	38 (59)	26 (41)
10	I have been encouraged to implement skin-to-skin contact with my infant	5 (8)	59 (92)

Mothers were first supported by a lactation consultant/breastfeeding counsellor within the first 8 h after delivery in 38% of cases, within the first 12 h in 23% of cases, within the first 24 h in 23% of cases and after 48 h in 16% of cases. Mothers began pumping milk within the first 24 h of delivery in 58% of cases, with 12% starting within the first 6–8 h. The mean frequency of milk pumping was 5.5 ± 1.6 times/day (items 11–13).

A previous positive breastfeeding experience was reported by 28% of the mothers (item 14). The majority of mothers (86%) declared that prior to delivery, they had decided to breastfeed their infant, although only 48% of mothers were advised by health care professionals during pregnancy to breastfeed (items 15–16).

The majority of the mothers stated that they had received information on how to identify hunger cues in their infant, whereas information on evaluation on how the infant was doing at suckling their breast was provided only to 50% of the mothers (Table 3). Breast milk was first fed by bottle and at breast in 78 and 22% of infants, respectively (item 19).

**Table 3:** Answers to the questions investigating the information received by the mothers during their hospital stays on recognition of infants’ hunger and evaluation of infant’s feeding at breast

Item n		Mothers (*n* = 64)	
		YES	NO
17	Did you receive information on how to recognize signs of hunger in your infant?	52 (81)	12 (19)
18	Did you receive information on how to evaluate how the infant suckled your breast?	32 (50)	32 (50)

A total of 37 (58%) mothers responded to the open-ended questions. The main comments that were stated by the mothers in answering the open-ended questions are reported in Table 4. The importance of breastfeeding support by health care professionals was emphasized by 40% of the respondents. Being close to their infant and the implementation of skin-to-skin contact were rated as interventions facilitating breastfeeding by 18% of the respondents, followed by the availability of a breast pump for milk production. The importance of being informed by health care professionals on how to get the baby latched on properly and how to identify hunger cues in the infant was also reported as being important in facilitating breastfeeding, as was breastfeeding peer support. In 3% of cases respondents indicated two or more interventions facilitating breastfeeding.

**Table 4:** Items/interventions facilitating and inhibiting breastfeeding according to mothers’ opinions

	% of mothers that responded to the open-ended questions (*n* = 37)
Items/Interventions Facilitating Breastfeeding	
Breastfeeding support by health care professionals	40%
Being close to my infant, opportunity for implementing skin-to-skin contact	18%
Breast pump available	12%
Breastfeeding peer support	9%
Being informed on how to position the infant at the breast and how to recognize signs of hunger in my infant	9%
Being informed on how to treat plugged ducts or mastitis	9%
Items/Intervention Inhibiting Breastfeeding	
Prematurity and/or low birth weight and/or presence of comorbidity	39%
Maternal stress and anxiety regarding infant’s clinical condition	18%
Pain and difficulty during milk pumping	16%
Lack of breastfeeding support by health care professionals	6%
Having multiples	5%

Among the factors that were reported to inhibit breastfeeding, having an infant born preterm and/or with low birth weight and/or affected by a comorbidity was reported by 39% of the mothers. Accordingly, maternal stress and anxiety regarding their infant’s clinical condition were also indicated as a barrier by 18% of the mothers. Pain and difficulty during milk pumping and a lack of breastfeeding support by health care professionals were also reported in 16 and 6% of cases, respectively, followed by having twins. In 6% of cases respondents indicated two or more interventions inhibiting breastfeeding.

Of the items on the questionnaire, only having experienced difficulties in pumping breast milk (item 8; OR = 4.6; CI 1.5–13.9, *p* < 0.007) and having experienced difficulties in providing an adequate amount of milk to the infant (item 9; OR = 3.57; CI 1.1–11.5, *p* = 0.033) were associated with a higher risk of the infant being fed with formula only. Having initiated milk expression after the first 6–8 h after delivery tended to be associated with a higher risk of being exclusively formula fed at discharge (OR = 1.59; CI 0.95–2.67, *p* = 0.075).

The retrospective power calculation showed that a total of 64 participants provides a power of 80% in order to estimate the mean of positive answers to items 1–10 (SD = 17) with 95% confidence interval no wider than 9%.

## Discussion

The present findings indicate that the majority of mothers perceived a supportive breastfeeding environment during their infant’s hospital stay. Additionally, most mothers stated they gained satisfaction with their breastfeeding experience and felt confident in sharing information with well-trained health care professionals about breastfeeding. These findings are consistent with previous data published in the literature. The importance of the relationship between parents and health care professionals, in terms of consistent breastfeeding support and education, has been reported as one of the main factors that facilitates breastfeeding [5, 14].

Notably, almost all mothers declared that they felt that feeding their infant human milk was beneficial for the infant’s health, suggesting that supplying human milk may represent a way in which mothers feel they contribute to the well-being of their infant. Accordingly, Alves et al. [15] investigated parents’ opinions on the facilitators of and barriers to human milk feeding in the NICU and found that 51.4% of the participants reported the beneficial health effects of human milk as one of the factors promoting breastfeeding. Indeed, the provision of mother’s milk allows the mother and her infant to be physically and emotionally connected. As a result, the mother takes part to her infant’s care and is supported in the achievement of the maternal role [15, 16].

In the present study, having been encouraged to implement skin-to-skin contact with their infant and having received information on how to identify their infants’ hunger cues were reported by the majority of mothers, further suggesting that they were supported in taking care of their infant within a family centre care context. Skin-to-skin contact has also been widely recognized as a significant contributor to the establishment and promotion of long-term breastfeeding of preterm infants [17, 18]. The importance of educating the mother on understanding signs of hunger in her infant has been highlighted by the implementation of the “cue-based” oral feeding approach in the NICU. This approach, which includes the evaluation of infants’ readiness for feeding and the identification of stress signals that arise during feeding, endorses achievement of independent feeding and makes infants’ feeding experiences as positive as possible [19, 20].

The rate of any breastfeeding at discharge in the present study was 66%, which is in accordance with previous data published in Italy [21, 22], suggesting an overall NICU environment supportive of supplying human milk to preterm infants. This is also supported by the fact that the mothers reported that the NICU resources available to assist them with breastfeeding were adequate. Consistent with our findings, Wilson et al. [23] performed a European collaborative survey and found that 58% of the preterm infants were fed any human milk at discharge, with large variations between regions.

However, it has to be considered that, in the present study, any infant was exclusively fed directly at the breast; the majority of infants, although fed human milk, consumed it by bottle. Accordingly, the majority of the mothers stated that their infant’s first human milk feeding was given by bottle, and only one out of every two mothers received information on how to evaluate infant feeding at the breast. In line with these results, Pineda et al. [24] found that nearly half of their 66 extremely preterm infants was never directly breastfed during hospitalization. This finding highlights not only the challenges faced in breastfeeding preterm infants but also the relative lack of promotion of direct breastfeeding, even though direct breastfeeding behaviours in the NICU have been positively associated with breastfeeding success at discharge and longer breastfeeding duration [24–26]. In addition, previous studies have indicated that human milk should not be fed by bottle in the NICU if mothers are willing to establish exclusive breastfeeding [25, 26]. The priority and effectiveness of direct breastfeeding support in NICU has been pointed out by Maastrup et al. [27] who demonstrated that most enrolled preterm infants achieved exclusively direct-breastfeeding at discharge, a figure that is consistent with the 51% reported by Briere et al. [26] in a study conducted retrospectively including 88 infants with a gestational age lower than 34 weeks.

In the present study, the majority of mothers had decided prior to or during pregnancy to breastfeed their infant, whereas less than half of the mothers declared they had been advised by health care professionals during their pregnancy regarding breastfeeding. These results highlight the importance of mothers’ prenatal intentions to breastfeed, which are predictive of positive breastfeeding outcomes when associated with the provision of appropriate support [28]. However, these findings also indicate the relative lack of provision of maternal prenatal education on the importance of breastfeeding as an effective intervention for health and development, although the importance of health care professionals supporting feeding decisions at key moments is widely acknowledged [28]. With regard to the perceived barriers to breastfeeding, 31% of the mothers reported that they experienced some obstacles to breastfeeding. Specifically, mothers who experienced difficulties in pumping breast milk and in providing an adequate amount of breast milk to their infant were more likely to not feed their infant with human milk at discharge. Accordingly, the early postnatal period of secretory activation in the mammary gland and coming to volume has been recognized as crucial in promoting human milk feeding [29]. Furthermore, when initiation of breast milk production takes place later than the first 6–8 h after delivery, formula feeding at discharge tends more likely to occur. Having an infant born very prematurely and/or with a low birth weight and/or affected by complications of prematurity was also reported as a barrier to breastfeeding in almost half of the interviewed mothers. Accordingly, maternal anxiety regarding their infant’s clinical condition was also reported as one of the factors inhibiting breastfeeding. These findings are in agreement with previously published data. Alves et al. [15] found that difficulties with milk production in association with worries regarding an inadequate milk supply were perceived by parents as being among the main barriers to breastfeeding. Boucher et al. [8] investigated the breastfeeding perceptions in 10 mothers that had their preterm infant hospitalized in the NICU for a minimum of 5 days. Mothers described their breastfeeding experiences and emphasized their worries regarding the adequacy of milk production in relation to the infant’s high demands. Furthermore, within the context of the NICU, which represents a particularly stressful environment for parents since they are physically separated from their infant, the lack of privacy, the provision of a structured feeding schedule, and the exhaustion and anxiety during infant’s hospitalization can contribute to reductions in the maternal breast milk supply [5, 8, 15]. Indeed, maternal anxiety and preterm birth are known to negatively affect lactogenesis [30], leading to a potential reduction in the breast milk supply. With regard to human milk production, it has previously been reported an association between timing of pumping initiation and volumes of milk. Specifically, at 3 weeks after delivery, volumes of milk resulted to be higher in women who had begun pumping within an hour of birth in comparison to those who had begun at 6 h after delivery [31]. Taken together, these findings highlight the importance of providing maternal education on milk expression and breastfeeding techniques, positively reinforcing a mother’s motivation and allowing parents to take part to the care of their infants in order to reduce distress and anxiety [5].

Moreover, the present results underline the need for providing a supportive breastfeeding environment in NICUs, within a family centre care context, focusing on the support of breast pumping mothers in the early lactation period and the promotion of direct breastfeeding.

This study has some limitations, since the experiences reported by the mothers interviewed in this study, who were relatively older and well educated, may not apply to all mothers who experience a premature delivery. Second, the study assessed the experience of mothers who delivered preterm infants requiring admission to level III care in one single institution; hence, the results could have been affected by the breastfeeding support provided in our NICU and by the structural characteristics of this NICU itself.

## Conclusions

The results of the present study allow further insight into the maternal experience with regard to the main facilitators of and barriers to supplying human milk during NICU hospitalization of preterm infants and indicate that health care professionals should modify their hospital policies in order to address known barriers to breastfeeding and endorse breastfeeding in mothers of preterm infants.

## Abbreviations

CI: Confidence Interval
NICU: Neonatal Intensive Care Unit
OR: Odds Ratio
